# Chronic Back Pain Diagnosed as Giant Osteoid Osteoma of the Thoracic Vertebra: A Case Report

**DOI:** 10.22114/ajem.v0i0.213

**Published:** 2019-07-12

**Authors:** Mohaddeseh Azadvari, Sarvenaz Rahimi, Seyede Zahra Emami Razavi

**Affiliations:** Physical Medicine & Rehabilitation Department, Imam Khomeini Hospital, Tehran University of Medical Sciences, Tehran, Iran.

**Keywords:** Back Pain, Osteoma, Osteoid, Spine, Thoracic Vertebrae

## Abstract

**Introduction:**

Diagnosing an osteoid osteoma as a benign tumor can be challenging owing to its different presentation patterns, ambiguous radiological findings and unusual sites of involvement. The present case report involves a 30-year-old female patient with a large osteoid osteoma of the thoracic vertebrae as an uncommon site of its presentation.

**Case Presentation:**

The patient presented with a one-year history of progressive right-sided upper back and interscapular pains. She was identified as a candidate for surgery using the whole body bone scan and a multiple detector computed tomography (MDCT) scan. A large 25-mm osteoid osteoma of the lamina of the third thoracic vertebra (T3) was also diagnosed through histopathology.

**Conclusion:**

As a potential cause of persistent back pain in young adults, an osteoid osteoma may be easily missed by routine radiographs. The CT scan is an effective tool in the investigation of the size and location of this tumor. Surgical excision can also be used for treating spinal lesions.

## Introduction

An osteoid osteoma is a benign osteoblastic tumor that normally develops in long bones and rarely in the thoracic spine (1, 2). This painful solitary bony lesion is much more prevalent in males, especially children and young adults aged 5–30 years (3). Pains, especially nocturnal ones, are the most common symptoms that can persist for quite a long time if the condition remains undiagnosed (4). In case of the failure of routine radiographs, more accurate disgnotic imaging techniques will be required to diagnose osteoid osteoma. Radionuclide bone scans and CT scans are useful tools for identifying and precisely locating the nidus. On the other hand, surgical excision of the nidus is recommended in refractory conditions of the disease. The present report involved a case of a large osteoid osteoma of the thoracic vertebrae as the abnormal site of its presentation.

## Case presentation

**Figure 1:**
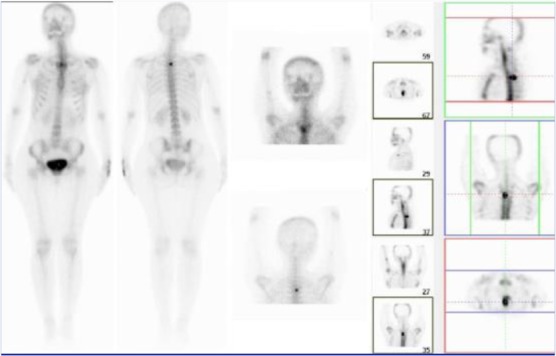
Whole body bone scan of the patient

**Figure 2:**
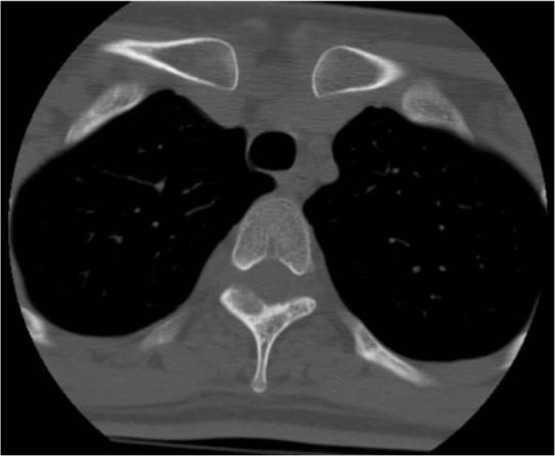
Multiple detector computed tomography image

**Figure 3:**
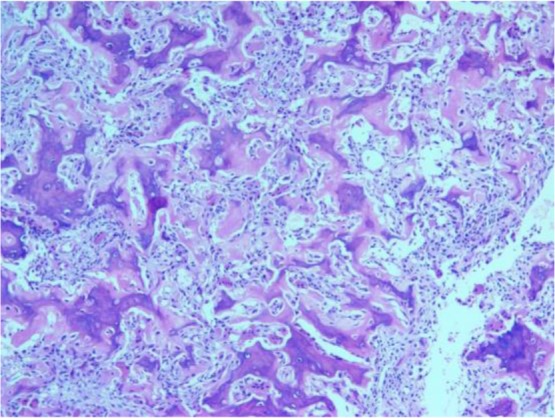
Histopathological findings of the patient

A 30-year-old female presented with a one-year history of progressive right-sided upper back and interscapular pains with no history of fever, night sweating, trauma and radicular pains. She reported exacerbating conditions at night along with coughs and sneezes. She still complained about the pain despite using medicines such as aspirin; nevertheless, her partial response to nonsteroidal anti-inflammatory drugs had positively affected her sleep patterns. Moreover, the magnetic resonance imaging scan of her thoracic spine performed one year before was reported as normal. Different evaluations ultimately diagnosed her with myofascial pain syndrome. Depo-Medrol plus lidocaine injected into a right rhomboid muscle failed to relieve the symptoms. Four months after the injection, she presented to the physical medicine clinic with severe upper back pains and anorexia-associated weight loss. She reported no history of serious health conditions, including chronic infections, metabolic diseases and malignancies, in either herself or her family. Furthermore, examinations suggested neither deformities nor apparent abnormalities. Her muscle bulk and tone were also found to be normal. A point of tenderness was, however, detected in the midline thoracic spine at the level of T3. Neurological examinations were also unremarkable. Moreover, routine laboratory tests suggested no abnormalities. The patient was then referred to an infectious disease specialist for further workup for mycobacterium tuberculosis, and all the results were reported as normal. The whole body bone scan showed an increased uptake at T3 (figure 1). In the next step, multiple detector computed tomography (MDCT) scan was ordered, and it revealed osteolytic lesions in the right lamina, suggesting osteoblastoma (figure 2). The patient underwent surgery and laminectomy. During the operation, the surgeon observed a 25-mm mass involving laminae and facet joints. Histopathological findings confirmed the diagnosis of osteoid osteoma (figure 3). The patient was found completely pain-free without any evidence of recurrence over the six-month follow-up.

## Discussions

An osteoid osteoma is a benign tumor that is commonly found in young patients. The lesion was first described by Jaffe in 1935 as a true neoplasm and histologically as a dense sclerotic overgrowth of bone with a highly-vascularized nidus containing varying levels of lymphocytic infiltration (5). Osteoid osteomas and osteoblastoma are histologically the same, with size being their only difference, as the latter is over 2 cm and the former normally below 2 cm. Osteoid osteomas can affect the uncommon site of the spine in only 1% of all spinal tumors. The usual sites involved respectively include the posterior elements of the lumbar spine (60%), cervical regions (27%) and the thoracic spine (12%) (6). In addition, transverse process involvement was reported in none of the recent 19 patients with spinal osteoid osteoma (7).

The condition often remains undiagnosed for at least six months after the emergence of the symptoms. The small size of the lesion, the complexity of spinal anatomy and the overlapping areas of soft tissue shadows usually cause routine radiographs to fail to pick up spinal osteoid osteoma. A CT scan is the best visualization technique for the nidus, and can exactly delineate the origin, size and location of the tumor, and helps with planning surgical excision (8, 9). Histopathological findings suggest that this tumor comprises a highly-vascularized nidus. Detecting a large osteoid osteoma 25 mm in size in the transverse process distinguished the present case report from other reports.

## Conclusions

As a potential cause of persistent back pain in young adults, a spinal osteoid osteoma may be easily missed by routine radiographs, and a high degree of clinical suspicion is required if it is not associated with scoliosis or neurological deficit. The CT scan is an effective tool in the investigation of the size and location of this tumor. Surgical excision can also be used for treating spinal lesions.
